# First dermoscopic characterization and histopathological correlation of panfolliculoma: a case report and review of the literature

**DOI:** 10.3389/fmed.2026.1836568

**Published:** 2026-05-28

**Authors:** Xiao Chen, Xindi Wang, Jianglin Zhang, Chen Liu, Zhaojun Sun, Youyou Zhou

**Affiliations:** 1 Department of Dermatology, Shenzhen People’s Hospital (The First Affiliated Hospital, Southern University of Science and Technology, The Second Clinical Medical College, Jinan University), Shenzhen, China; 2 School of Medicine, Southern University of Science and Technology, Shenzhen, Guangdong, China

**Keywords:** dermoscopy, hair-derived skin tumor, histopathology, panfolliculoma, skin adnexal tumor

## Abstract

Panfolliculoma (PF) is a rare benign cutaneous adnexal neoplasm exhibiting panfollicular differentiation, frequently misdiagnosed as basal cell carcinoma (BCC) due to overlapping clinical features. We present the case of a 77-year-old woman with a slow-growing nodule on the nasal tip, initially suspected to be a BCC. Definitive diagnosis of cystic PF was established through histopathological evaluation, while novel dermoscopic characterization revealed characteristic keratin-filled follicular openings and peppercorn-like granules alongside BCC-like vascular structures. This report reviews the clinical characteristics, dermoscopic findings, and histopathological features of PF based on our case and a systematic analysis of 54 globally reported cases. Identifying specific dermoscopic markers of follicular lineage can help distinguish PF from malignant mimics, thereby guiding appropriate clinical management and avoiding unnecessary aggressive surgical interventions.

## Introduction

Panfolliculoma (PF) is a rare cutaneous adnexal neoplasm with prominent follicular differentiation. As a hair-derived benign tumour, PF exhibits a unique feature in skin histopathology: It can differentiate into any component of the hair follicle, including the upper segment (infundibular and isthmic) and the lower segment (including the hair shaft, bulb, and hair matrix). Its typical clinical manifestations are slow-growing cystic nodules or dome-shaped nodules similar to cysts, and it has been reported that PF can mimic basal cell carcinoma (BCC) clinically (1). To the best of our knowledge, the previously reported cases described the dermoscopic features of PF, which remain largely undocumented. In this study, we report a rare case of PF that is highly similar to BCC, both clinically and dermoscopically. In addition, we analyzed published case reports to summarize the clinical features, dermoscopic and histopathological features, and differential diagnosis of PF.

## Case description

**Figure 1:**
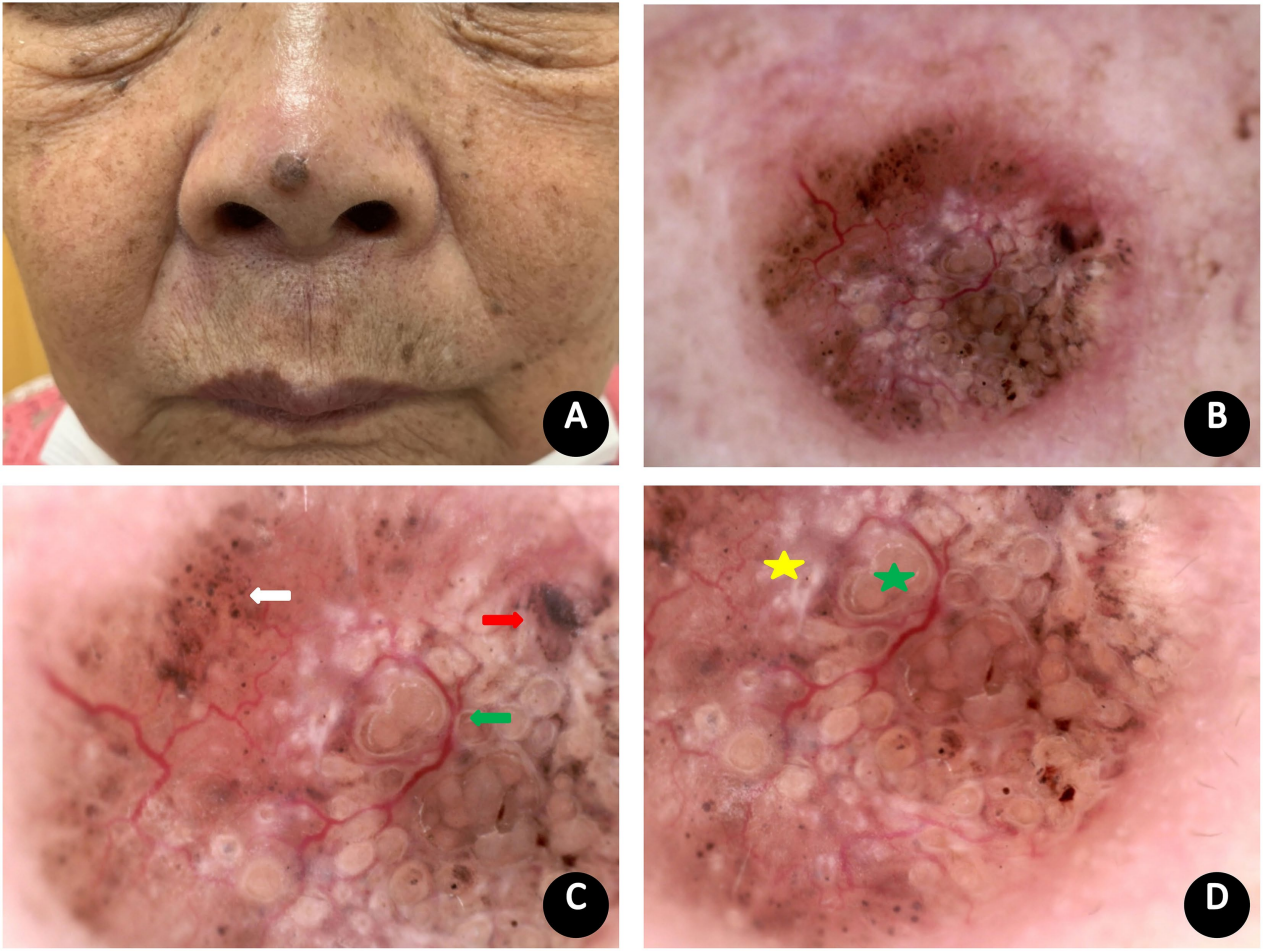
Clinical signs and dermoscopic images. **(A)** A brownish round tumor on the tip of the nose, slightly elevated, with a smooth surface, hard texture, and well-defined margins. **(B)** Dermatoscopic examination: pale brown background, keratin-filled follicular openings visible, multiple grey-brown lobules, brown ovoid structures, thick dendritic vessels (25×). **(C)** Multiple grey-brown lobules (white arrow), brown ovoid structures (red arrow), and thick dendritic vessels (green arrow) (55×). **(D)** Expanded keratin-filled follicles with pearl-like structures (green star), white structureless areas, and white streaks (yellow star) (55×).

**Figure 2:**
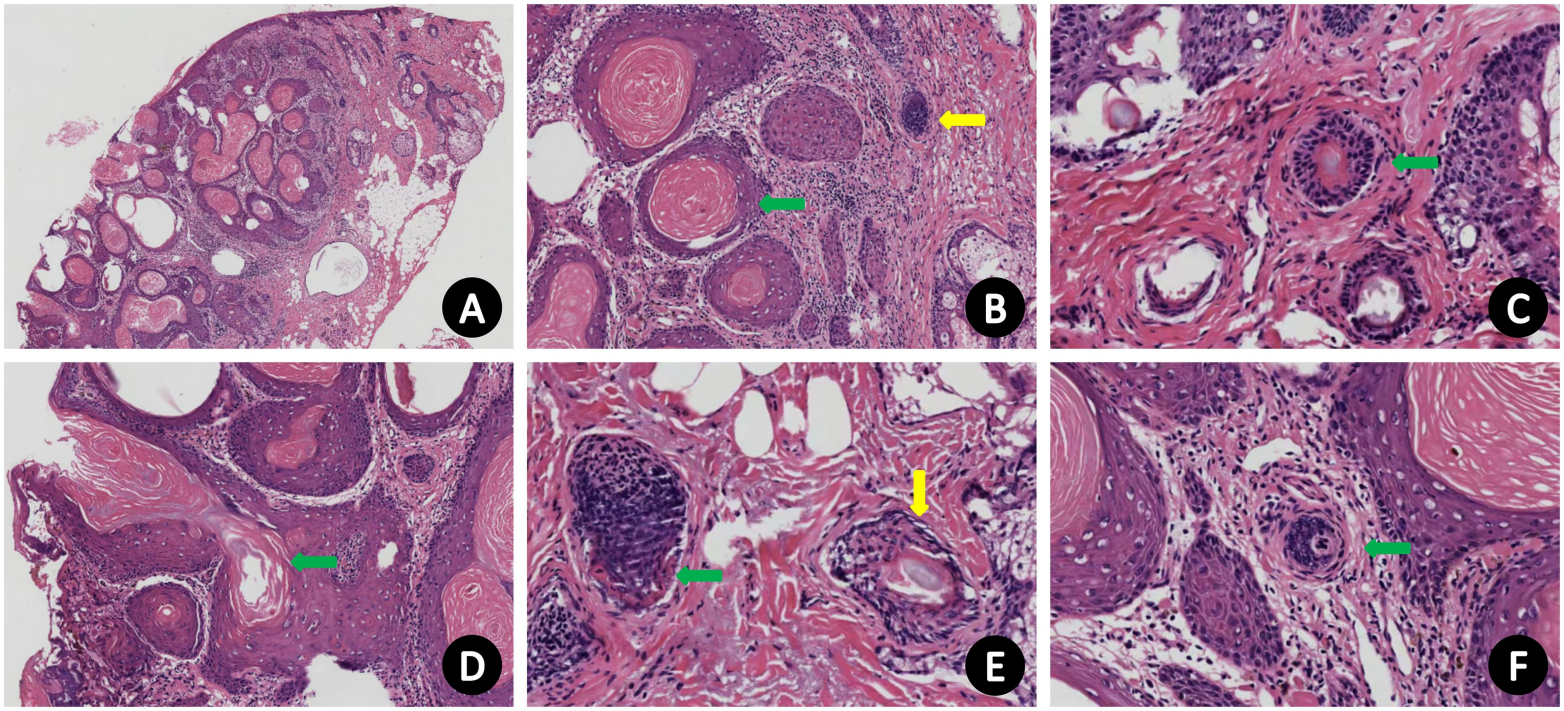
Histopathologic picture. **(A)** The tumor is well-defined, with numerous islands of dilated, keratin-filled cells in the dermis and some microcysts opening at the skin surface (hematoxylin and eosin [H&E], 10x). **(B)** Microcysts lined with stratified squamous epithelium (green arrow), with follicular germinal cells clustered to form substantial palisaded islands (yellow arrow) (H&E, 40x). **(C)** Island of cells consisting of eosinophilic trichohyalin granules and blue–grey keratinocytes, with peripherally fenestrated basophilic cells (green arrow) (H&E, 100x). **(D)** Granular layer disappears, visible as islands of hyaline protein-rich cells with central eosinophilic keratin (green arrow) (H&E, 100x). **(E)** Interpapillary plastid formed by aggregations of basophilic cells (green arrow). Cell islands composed of germinative cells, matrix, etc. (yellow arrow) (H&E, 100x). **(F)** Hair bulbs encircling papillae (green arrow) (H&E, 100x).

**Figure 3:**
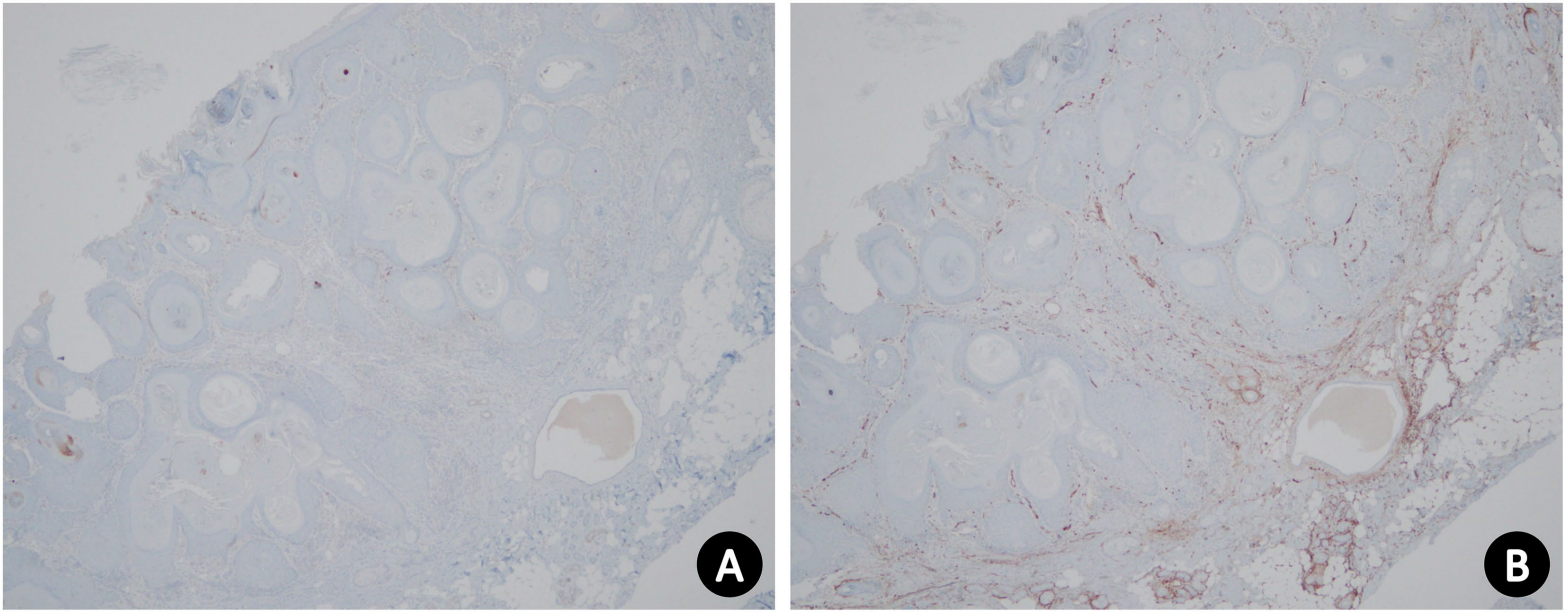
Immunohistochemical images. **(A)** BerEP4 stained negative (SP, 40x); **(B)** CD34 staining shows positive mesenchymal vessels but negative tumor (SP, 40x).

We report the case of a 77-year-old woman who presented to the dermatology department with a 3-year history of a brown mass on the nasal tip. Dermatological examination revealed a slightly elevated, brownish, round tumour on the nasal tip measuring approximately 0.7 cm × 0.7 cm. The lesion had a smooth surface, firm texture, well-defined margins, and moderate mobility. It was non-tender and showed no signs of erythema, swelling, or ulceration (Figure 1A). Her past medical and family histories were unremarkable. Dermatoscopic examination revealed a pale brown background, with dilated follicular openings/keratin-filled follicular openings visible, white to yellowish-white structureless areas, white streaks, multiple grey–brown lobules, brown ovoid structures, and thick dendritic vessels. Dermoscopic measurement showed that the lesion was approximately 0.96 cm × 0.83 cm. These findings do not rule out BCC (Figures 1B–D). Histopathological examination of the lesions showed a well-defined intradermal neoplasm containing numerous islands of dilated keratin-filled cells, with some microcysts opening at the skin surface (Figure 2A). Cystic structures with cystic walls composed of a stratified squamous epithelium were observed, representing infundibular differentiation (Figure 2B, green arrow). Peripheral palisading follicular germinative cells formed substantial islets, representing follicular differentiation (Figure 2B, yellow arrow). Cell islands composed of eosinophilic trichohyalin granules and blue–grey keratinocytes, with peripherally fenestrated basophilic cells, indicated differentiation towards the inner root sheath (Figure 2C, green arrow). Disappearance of the granular layer, the presence of transparent cell islands, and visible eosinophilic keratin represented outer root sheath differentiation (Figure 2D, green arrow). Cell islands were composed of germinative cells and matrix with keratin and hair stalk-like structures in a cystic structure, representing hair differentiation (Figure 2E, yellow arrow) (2, 3). Interpapillary plastid were formed by aggregations of basophilic cells (Figure 2E, green arrows), and hair bulbs encircled papillae (Figure 2F, green arrow). No obvious cellular anisotropy was observed, and no artificial lacunae or mucus-like interstitial changes were observed in the section. An immunohistochemical staining showed that the tumour was negative for BerEP4 (Figure 3A) and CD34 (Figure 3B), whereas CD34 staining showed a positive expression of interstitial vessels. At the final diagnosis, a panfolliculoma cyst was noted. The patient had no recurrence after surgical removal of the tumour and is currently under continuous follow-up in our department.

## Methods

A comprehensive literature search was conducted using databases, including PubMed, Web of Science, and the China National Knowledge Infrastructure (CNKI), from their inception to 9 March 2026. The search terms used were “panfolliculoma” OR “cystic panfolliculoma” OR “superficial panfolliculoma” OR “nodular panfolliculoma.” References of the retrieved articles were also manually screened to identify additional cases. A total of 55 confirmed cases (including the present case) were ultimately included in our statistical analysis.

## Results

We found that 28 panfolliculoma-related papers were retrieved (1–28), reporting 55 cases (including our reported cases). One case was excluded from the primary clinical and histopathological analyses, as the original report lacked essential patient demographics (age and gender) and failed to specify the histopathological subtype or clinical morphology. Notably, the total number of evaluated cases varies slightly among different parameters depending on the availability of specific data in the original reports.

PF mostly presents as pink or skin-coloured nodules, with the age of onset ranging from 8 to 88 years. The highest incidence was observed in the older adults (>60 years old) (25/54), followed by quinquagenarians (50–60 years old) (11/54), and can also occur in adults and middle-aged individuals (25–49 years old) (13/54). The condition was less frequently observed in young people aged 18–24 years old (3/54) and was rare in adolescents and children under 18 years old (2/54) (Supplementary Figure S1A). A slight male predominance was noted (30/54), with a male-to-female ratio of approximately 1.25:1. Lesions were most commonly located on the head and neck (37/54), particularly the facial area (18/54), followed by the scalp (14/54), the ears (4/54), and the neck (1/54). They were also found on the extremities (11/54) and the trunk (6/54) (Supplementary Figure S1B). Tumour sizes ranged from 0.3 cm x 0.3 cm to 6 cm x 4 cm, typically presenting as slowly growing nodules. Regarding initial clinical impressions, 48 cases had available preliminary diagnoses. PF was most frequently misdiagnosed as a cyst, such as an epidermoid cyst or follicular cyst (17/48). It was also easily misdiagnosed as basal cell carcinoma (16/48), squamous cell carcinoma (7/48), and miscellaneous skin lesions such as lipomas, lymphangiomas, seborrhoeic keratoses, warts, melanomas, or unspecified tumours (8/48). Six reported cases were not included in the statistics because no initial clinical diagnosis was provided. (Supplementary Figure S1E). Histopathological evaluation (available for 53 cases) revealed cystic PF most commonly (23/53), followed by superficial PF (13/53) and nodular PF (12/53). Less common variants included endophytic PF (3/53), PF with sebaceous differentiation (1/53), and mixed nodular and cystic PF (1/53) (Supplementary Figure S1C). Furthermore, geographic distribution analysis indicated a higher prevalence of reported cases in Western countries, including Europe and the United States (42/55) than in Asia (13/55), of which 8 of the Asian cases originated from China (Supplementary Figure S1D).

## Discussion

First described by Ackerman et al. in 1993, PF is a rare benign cutaneous adnexal neoplasm exhibiting panfollicular differentiation (29). According to Juang et al. (4), as of 31 March 2022, only 18 publications reporting 43 cases of PF has been reported worldwide. Our comprehensive literature review identified an additional 12 cases. Therefore, the case reported in this study represents the 55th reported case of PF worldwide and, to the best of our knowledge, the first to feature a detailed dermoscopic characterization.

PF is a benign tumour capable of differentiating towards any component of the hair follicle, encompassing the upper segments (infundibulum and isthmus) and the lower segments (hair shaft, bulb, and hair matrix). The mass presents as solid, cystic, or cystic-solid nests. Based on the histopathological classification proposed by Shan and Guo, PF has been classified into three types: nodular panfolliculoma (NPF), superficial panfolliculoma (SPF), and cystic panfolliculoma (CPF) (5); subsequently, another variant characterized by an endophytic structure of the hair follicle (differentiation towards sebaceous glands) has also been reported (6, 7). In NPF, the tumour cells form large clusters, primarily in the dermis; in SPF, the tumour cells are primarily located in the superficial layers of the epidermis and/or dermis; and in CPF, the tumour cells are confined to the capsule wall or protrude from the capsule wall (7). The case we report belongs to the CPF subtype. The pathohistological examination showed that the tumour was a cystic structure located within the dermis with differentiation towards various components of the hair follicle, and the tumour cells were confined within the wall of the follicle, consistent with the presentation of a benign tumour. Immunohistochemical staining showed positive CD34 staining for mesenchymal vessels but negative for the tumour itself for differential diagnosis with extranodal root sheath tumour. Furthermore, although BerEP4 staining was negative in our case, recent literature indicates that follicular germinative cells in PF can also exhibit positive BerEP4 expression. Consequently, differentiating PF from BCC requires a comprehensive assessment incorporating other histological criteria, such as the absence of retraction clefts that are characteristically observed in BCC.

Clinical information on PF was summarized based on reported cases. It was found that there is a slight gender difference in PF, with a slightly higher incidence in male individuals than in female individuals. It can occur at any age but is most common in middle-aged and older people. Lesions frequently occur not only on the head, face, and limbs but also on the trunk and are often solitary pink or skin-coloured nodules. The course of the disease is slow, generally without conscious symptoms, with no tendency to self-heal and no familial susceptibility. There may be regional variations, common in Europe and the United States. Patients with self-perceived abnormalities or co-infections often present to the clinic.

### Clinical differential diagnosis

The clinical differential diagnosis of PF includes hair cysts, epidermoid cysts (EC), basal cell carcinoma (BCC), and squamous cell carcinoma (SCC) (4, 5). Clinically, distinguishing PF from pigmented BCC can be particularly challenging in individuals with higher skin phototypes, such as the East Asian patient in this case (Fitzpatrick type IV). In these populations, active melanin deposition often masks the benign features of adnexal neoplasms, resulting in a clinical morphology that closely mirrors malignant lesions (30, 31). Both PF and pigmented BCC can present as slow-growing, well-defined, pigmented nodules on the head and face. However, while pigmented BCC may frequently exhibit central ulceration or pearly borders due to its invasive nature, PF generally maintains an intact epidermal surface (32). Given these overlapping clinical features, our case of PF closely resembled pigmented BCC, highlighting the critical need for dermoscopic evaluation.

### Dermoscopic differential diagnosis

To the best of our knowledge, dermoscopic manifestations of PF have rarely been documented in the available literature. The classic dermoscopic diagnostic model for pigmented BCC consists of one negative criterion, i.e., the absence of a pigmented nevus/no evidence of nevus cells, and six positive criteria, including large bluish-grey ovoid nests, multiple bluish-grey globules, maple-like patterns, spoke-wheel structure, ulceration, and dendritic vascularity. The diagnosis of pigmented BCC can be made by fulfilling one negative criterion and at least one positive feature (33). Dermatoscopy of the PF showed the classic diagnostic pattern of pigmented BCC: grey–brown ovoids, multiple grey–brown ovoids, multiple grey–brown nodules, and dendritic vessels. This mimicry is common in pigmented skin, where melanin accumulation can also resemble other pigmented entities such as pigmented pilomatricoma or trichoblastoma. However, some of the dermoscopic features of SCC can also be observed: a large number of target-like follicles/target-like follicles filled with keratin. Notably, under 55x magnification, these targetoid follicles exhibited an irregular, crateriform surface topography. In addition, pepper-like particles can be observed.

**Figure 4:**
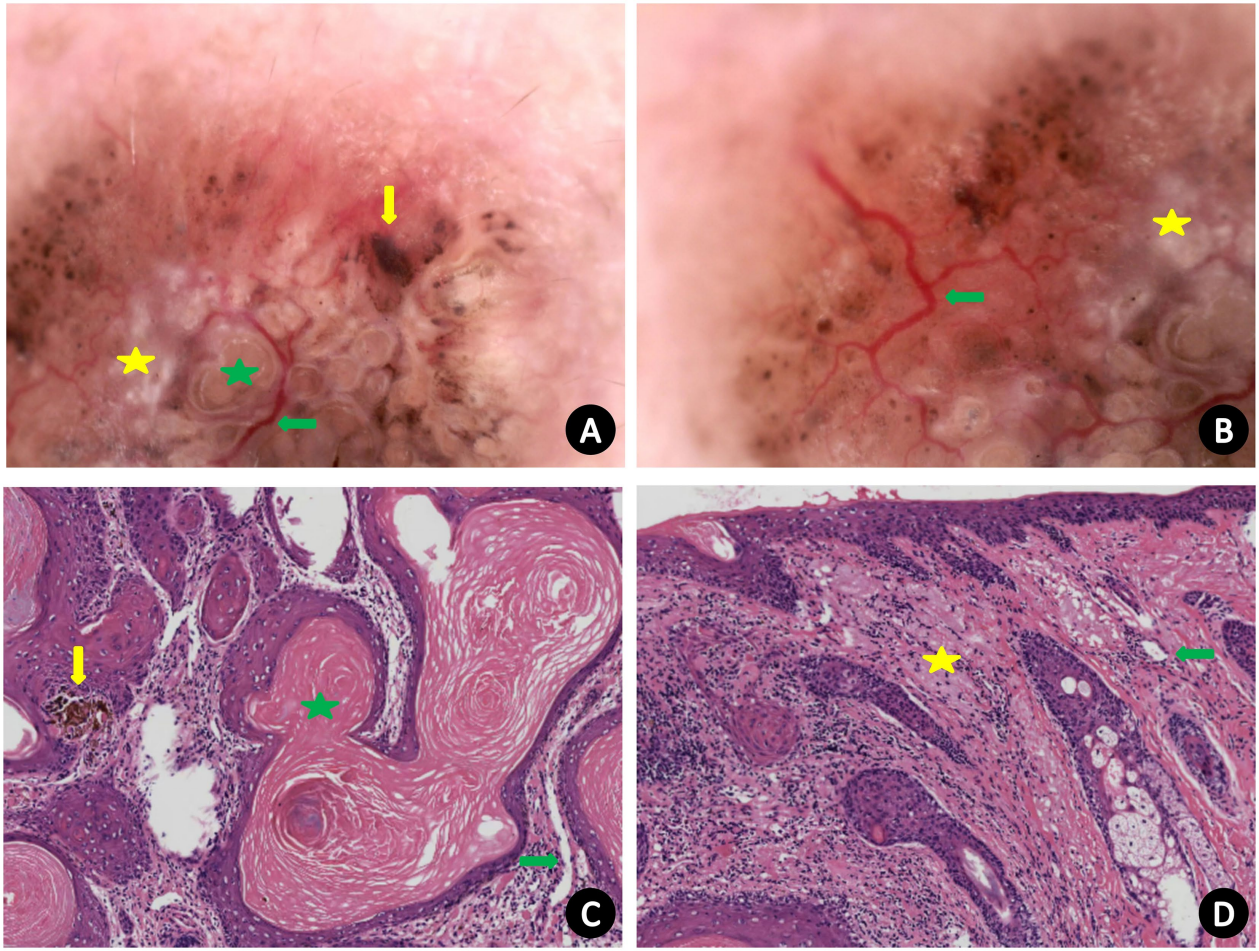
Correlation analysis between dermoscopy and histopathology. **(A)** Grey–brown ovoid structures (yellow arrowheads), white unstructured areas with white streaks (yellow stars), dilated follicular openings (green stars), and thick dendritic vessels (green arrows) (ELM, 55x). **(B)** Thick dendritic vessels (green arrows) and white unstructured areas (yellow stars) (ELM, 55x). **(C)** Pigment deposition (yellow arrowheads), expanded follicular funiculus (green star), and vessels (green arrows) (H&E, x40). **(D)** Degenerated elastic fibres (yellow stars) and vessels (green arrows) (H&E, x40).

Detailed clinicopathological correlation revealed the following associations: dermoscopic follicular keratin plugs (Figure 4A, green star) correspond to keratin-filled cystic structures in pathohistological sections (Figure 4C, green star) and microcystic structures opening into the epidermis as dilated follicular funnels. While these findings confirm a follicular origin, they also serve as critical discriminators from other benign adnexal neoplasms. Unlike trichoepithelioma, which typically displays multiple milia-like cysts and fine vessels, or trichoblastoma, which often exhibits blue-grey globules and arborizing vessels, the prominent keratin-filled openings and peppercorn-like granules observed in this study are distinct hallmarks of PF’s pan-follicular differentiation. The grey–brown ovoid structures and multiple grey–brown globules (Figure 4A, yellow arrowheads) correspond to pigment deposition (Figure 4C, yellow arrowheads), which may be related to pigment produced by the trichogenic tumour. White unstructured areas with white streaks (Figures 4A,B, yellow stars) correspond to degenerated elastic fibres (Figure 4B, yellow stars) associated with heliotrope elastoid changes in the patient’s nose. Thick dendritic vessels (Figure 4A, green arrows) correspond to dilated small vessels (Figures 4C,D, green arrows).

It is important to acknowledge that the dermoscopic features described in this study are based on a single case, and drawing definitive, generalized conclusions requires caution. However, based on the histopathological correlation, we hypothesize that the keratin-filled follicular openings and white-to-yellowish structureless areas directly correspond to the infundibular and isthmic differentiation inherent to PF, making them potentially reliable, specific diagnostic clues for follicular lineage. Conversely, the thick dendritic vessels and grey–brown lobules, while prominent in our case and mimicking BCC, may be less specific, likely reflecting general tumour angiogenesis and focal pigmentation. Crucially, identifying these lineage-specific markers allows for the differentiation of PF not only from BCC but also from other benign hair follicle tumours dermoscopically. For instance, trichoepithelioma typically presents with multiple milia-like cysts and fine, non-arborizing vessels, whereas trichoblastoma often exhibits blue–grey globules and arborizing vessels that can also closely mimic BCC. In contrast, the prominent keratin-filled follicular openings and peppercorn-like granules observed in our case serve as distinct hallmarks of PF’s panfollicular differentiation, setting it apart from adnexal tumours with more restricted developmental lineages.

Identifying such specific markers is vital when evaluating pigmented lesions in populations with higher skin phototypes, as it narrows the differential diagnosis beyond BCC to include other pigmented entities such as pigmented pilomatricoma or pigmented trichoblastoma. Furthermore, in addition to follicular neoplasms, PF is easier to distinguish from EC under dermoscopy, with EC appearing microscopically as acne-like openings or light brown/greyish-blue spirals, often described as the central pore sign, and a creamy white, bluish-grey, or greyish-yellow homogeneous background around the lesions (34). Hair cysts do not have characteristic dermoscopic features, and differentiation between the two is primarily based on histopathology. In addition, PF should be distinguished from other lesions, including seborrhoeic keratosis (SK), actinic keratosis (AK), which are dermatoscopic manifestations of these skin swellings: SK shows gyrus-like structures, fingerprint-like structures, sulcal-ridge structures, acne-like openings, cornified cysts, and hairpin vessels (35).

Although the acne-like opening of the PF is visible in this case, this structure is more appropriately described as an expanded follicular opening, and its vascular pattern is thick dendritic vessels, which is not consistent with the vascular pattern of SK. In addition, AK shows follicular target-like structures on dermoscopy, but with red, reddish-brown, or greyish-brown pseudo-reticulation in the background. Wavy, thin, dendritic blood vessels can be observed around the hair follicles, some with a strawberry-like appearance, called the strawberry sign. Pigmented AK is also noted as greyish-brown granules around hair follicles (annular-granular pattern or asymmetric pigmentation) (36).

### Histopathological differential diagnosis

Definitive differentiation relies on histopathology. The main histopathological differential diagnoses of PF primarily include trichofolliculoma, dilated pore of Winer, hair cysts, trichoblastoma, pilomatricoma, and BCC (4, 5, 27). Trichofolliculoma also differentiates into the various components of the hair follicle, making it the primary differential diagnosis that must be distinguished from CPF. In pathological sections, multiple secondary follicles may be seen radiating around the primary follicles. The tumour consists primarily of a central cystic cavity, which is keratinized in the infundibular region. Fully mature follicular structures are visible, suggesting more mature differentiation than CPF. In fact, trichofolliculomas are essentially hamartomas of the hair follicle. Dilated pore of Winer is characterized as a follicular funicular structure opening onto the skin surface, with radial finger-like projections visible at the periphery and invaginated epidermal acanthosis. Hair cysts lack a granular layer. There is abrupt cellular keratinization of the tumour capsule wall, and the capsule is filled with eosinophilic, dense keratinous contents. Trichoblastoma is a tumour mass dominated by basaloid cells with a mesenchymal component capable of inducing differentiation of basaloid cells into hair follicles; however, it lacks complete panfollicular differentiation (37). Pilomatricoma consists of trichoblasts and ghost cells that do not differentiate into other follicular structures. Crucially, when differentiating PF from BCC histopathologically, BCC is characterized by a predominance of basal cells forming basaloid nests with peripheral palisading, typical artificial retraction clefts between the tumour nests and the stroma, and mucin deposits. In contrast, PF exhibits true panfollicular differentiation and lacks the characteristic widespread retraction clefts of BCC. In addition, PF should also be distinguished from congenital nevus with trichofolliculoma, a rare nodular skin condition that also shows histopathological differentiation toward total follicular structures. However, it is a hamartoma that is present at birth, most commonly in children, and can be distinguished from an allantoic follicular tumour by the combination of clinical manifestations (27).

In summary, PF is a rare benign cutaneous swelling with clinical features: the incidence in men is slightly higher than that in women, primarily in middle-aged people and older adults. PF is characterized by usually pink or skin-coloured single nodules on the head, face, and limbs with slow progression. It is generally asymptomatic and is common in Europe and the United States. Currently, diagnosis is primarily based on histopathological observation of features differentiating various follicular components. Dermoscopy shows some of the features of BCC (multiple grey–brown lobules, brown ovoid structures, and thick dendritic vessels), keratin-filled follicular openings, and peppercorn-like granules. A single case report is insufficient to summarize the microscopic features of PF. Therefore, the dermoscopic features of PF need to be refined.

## Data Availability

The original contributions presented in the study are included in the article/supplementary material, further inquiries can be directed to the corresponding authors.

## Supplementary material

SUPPLEMENTARY FIGURE S1 Literature statistics. **(A)** Age distribution map (*N* = 54); **(B)** Site of lesion occurrence (*N* = 54); **(C)** Histopathological typing (NPF: nodular PF; CPF: cystic PF; SPF: superficial PF) (*N* = 53, as one case lacked detailed subtyping); **(D)** Regional distribution differences (*N* = 55, including one case with limited clinical data but known origin); **(E)** Clinical misdiagnosis classification (BCC: basal cell carcinoma; SCC: squamous cell carcinoma) (*N* = 48, as 6 cases lacked initial clinical impressions).
